# Growth and antioxidant responses to water stress in eggplant MAGIC population parents, F_1_ hybrids and a subset of recombinant inbred lines

**DOI:** 10.1186/s12870-024-05235-w

**Published:** 2024-06-15

**Authors:** Martín Flores-Saavedra, Mariola Plazas, Pietro Gramazio, Oscar Vicente, Santiago Vilanova, Jaime Prohens

**Affiliations:** Instituto de Conservación y Mejora de la Agrodiversidad Valenciana, Universitat Politècnica de València, Camino de Vera 14, Valencia, 46022 Spain

**Keywords:** Eggplant, Water stress, Breeding, Hybrids, MAGIC population, Oxidative stress

## Abstract

**Background:**

The generation of new eggplant (*Solanum melongena* L.) cultivars with drought tolerance is a main challenge in the current context of climate change. In this study, the eight parents (seven of *S. melongena* and one of the wild relative *S. incanum* L.) of the first eggplant MAGIC (Multiparent Advanced Generation Intercrossing) population, together with four F_1_ hybrids amongst them, five S5 MAGIC recombinant inbred lines selected for their genetic diversity, and one commercial hybrid were evaluated in young plant stage under water stress conditions (30% field capacity; FC) and control conditions (100% FC). After a 21-day treatment period, growth and biomass traits, photosynthetic pigments, oxidative stress markers, antioxidant compounds, and proline content were evaluated.

**Results:**

Significant effects (*p* < 0.05) were observed for genotype, water treatments and their interaction in most of the traits analyzed. The eight MAGIC population parental genotypes displayed a wide variation in their responses to water stress, with some of them exhibiting enhanced root development and reduced foliar biomass. The commercial hybrid had greater aerial growth compared to root growth. The four F_1_ hybrids among MAGIC parents differed in their performance, with some having significant positive or negative heterosis in several traits. The subset of five MAGIC lines displayed a wide diversity in their response to water stress.

**Conclusion:**

The results show that a large diversity for tolerance to drought is available among the eggplant MAGIC materials, which can contribute to developing drought-tolerant eggplant cultivars.

## Introduction

Amongst the abiotic factors that adversely affect crops, water stress stands out as a significant and persistent challenge in many areas, leading to significant reductions in agricultural productivity [1–3]. Despite advances in agricultural techniques, the detrimental effects of water stress have been partially mitigated by improvements in irrigation systems and efficient management practices [4, 5]. Nevertheless, changes in precipitation patterns observed in recent decades continue to reduce water availability, and more than two-thirds of the world’s population is expected to face water scarcity in the near future [6]. Given this predicament, there is an urgent need to identify and breed genotypes capable of sustaining optimal growth under deficit irrigation conditions [7].

Horticultural crops with high irrigation requirements [8] are particularly vulnerable to reduced precipitation, a scenario exacerbated by climate change [9]. In this context, eggplant (*Solanum melongena* L.) is emerging among vegetables as a promising candidate for cultivation under water-stressed conditions [10, 11]. A remarkable drought tolerance response has been observed in this species at the biochemical level, involving increased phenolic compounds and flavonoids to alleviate oxidative stress induced by water deficit [12]. It has also been observed that eggplant genotypes with rapid growth and extensive root development perform better under water stress [13]. On the other hand, introgression of genomic regions from the wild parent *S. incanum* into eggplant has led to improvements in water content, water use efficiency and chlorophyll content, improving tolerance to water stress [14].

The use of hybrid varieties has led to significant advances in agriculture, with the potential to increase production and resilience to biotic and abiotic stresses [15]. In eggplant, promising responses under water stress conditions have been identified through hybridization, with interspecific hybrids of *S. melongena* × *S. incanum* and *S. melongena* × *S. insanum* showing superior growth under limited water supply compared to their parents [16]. In addition, Multiparent Advanced Generation Intercrossing (MAGIC) populations are increasingly recognised as a powerful tool for plant breeding, facilitating the identification of genomic regions and the selection of recombinant lines with desired traits [17]. These populations have proven effective in locating quantitative trait loci (QTLs) and selecting water stress-tolerant materials in several crops, including maize [18], beans [19], and chickpeas [20]. In the case of eggplant, the first MAGIC population, which includes an accession of the drought-tolerant *S. incanum* amongst its eight parents [21], promises to be a valuable resource for improving water deficit tolerance in this crop.

The objective of this study is to investigate the variation in water stress tolerance and related traits amongst the eight parents and their four F_1_ hybrids used to develop the MAGIC population, as well as amongst a subset of genetically diverse MAGIC recombinant inbred lines under control and water stress treatments. The aim is to identify materials with contrasting performance under water stress and to assess the potential of the MAGIC population for breeding for increased tolerance to water stress.

## Materials and methods

### Plant material

A total of 18 genotypes were included in the present study. The plant materials included the eight parents from a multi-parent advanced generation intercross (MAGIC), seven of which were from *Solanum melongena* (accessions MM1597 (A), DH ECAVI (B), AN-S-26 (D), H15 (E), A0416 (F), IVIA-371 (G), ASI-S-1 (H)) and one from the wild relative *S. incanum* (accession MM577 (C)) [19], as well as four F_1_ hybrids amongst the eight parents (A × B, C × D, E × F and G × H) and a subset of five genetically distant MAGIC lines (M40, M45, M194, M204, M262). The five lines were selected from a total of 420 using a neighbour-joining tree based on genotypes [21] and selecting one line from each of the five main branches. The commercial F_1_ hybrid Petra (Semillas Fitó, Barcelona, Spain) was included to assess the response to drought of the MAGIC materials compared to a widely grown hybrid.

### Growing conditions

The seeds of the 18 genotypes were germinated in petri dishes according to a published protocol [22]. After germination, the seeds were placed in seedling trays with growing substrate (Humin substrate N3, Klasmann-Deilmann, Germany) in a growth chamber. When the plants reached the stage of two developed leaves (46 days after sowing), they were transferred to a controlled temperature benched greenhouse (maximum 30ºC and minimum 15ºC) and transplanted into 1.3 L pots with the same substrate as the seedlings and fertilised with 200 ml of Hoagland solution [23]. Fifteen homogeneous plants from each genotype, corresponding to five replicates for the baseline measurements at the start of the treatments, as well as for each of the control and water stress treatments, were used. The pots were randomly distributed on the greenhouse benches.

The treatments consisted of watering the pots every two days to 100% (control) and 30% (water stress) of the substrate field capacity (FC). The level of irrigation required to induce water stress in plants was determined in a previous study (unpublished data). This was done using the gravimetric method [24] by weighing each pot and watering it with the appropriate quantity of water to reach the target FC. To determine the weight of the pots at 100% and 30% FC, six pots were watered to saturation with a dry substrate content equivalent to 148 g. Subsequently, covering the top to prevent evaporation, they were left to drain for 48 h. The weight of the pot after this period was considered to be 100% FC. After subtracting the weight of the pot and the dry substrate, it was found that 100% FC was reached with 651 g of water, whereas for the water stress treatment, 195.3 g of water were required to reach 30% FC.

**Fig. 1:**
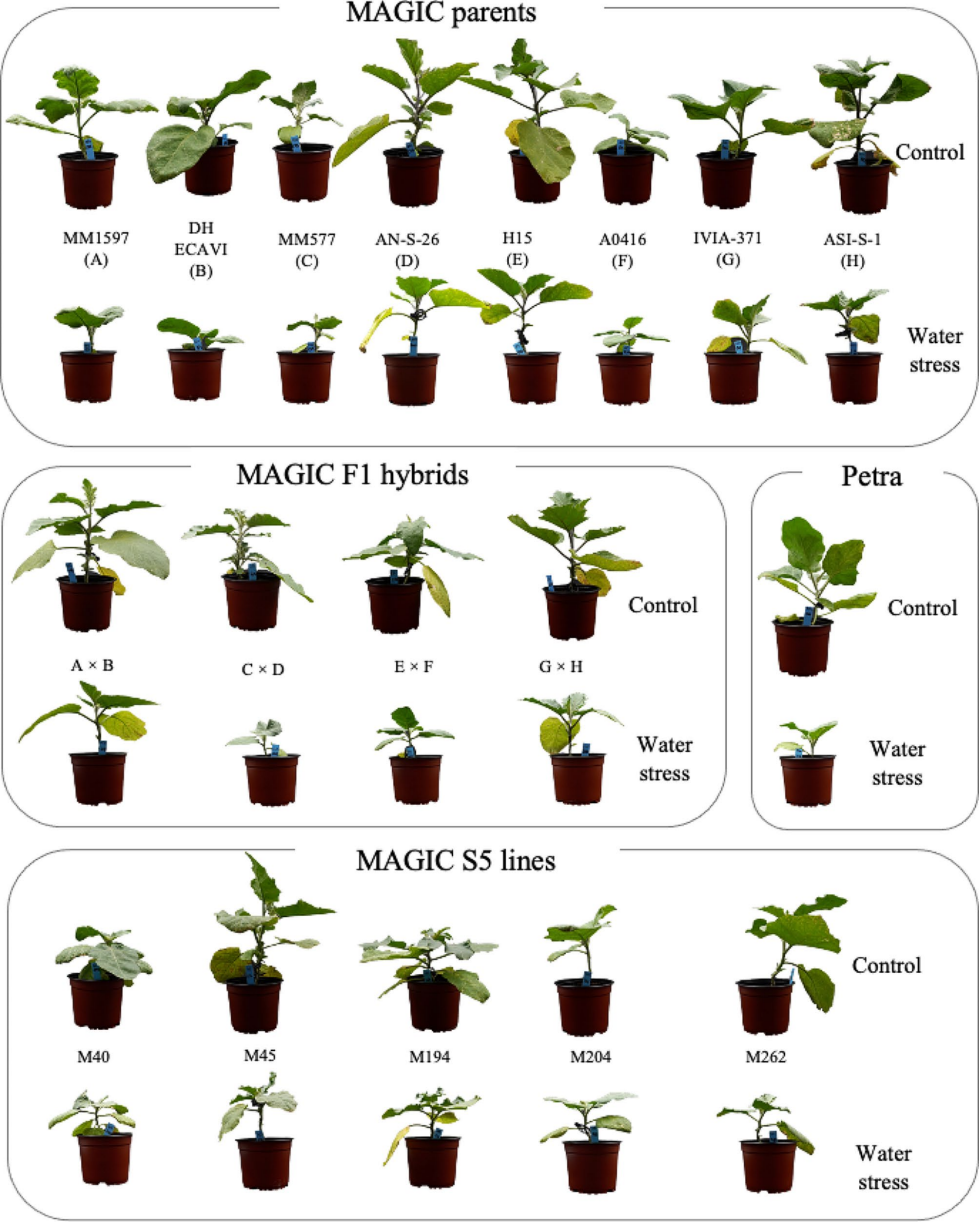
Eggplant MAGIC materials (parents, F_1_ hybrids and a subset of S5 lines) and an F_1_ commercial hybrid (Petra) after 21 days of irrigation treatments at 100% (control) and 30% (water stress) of field capacity

Three weeks after transplanting, before starting the treatment, a baseline assessment of growth and biomass was made on five plants of each genotype (67 days after sowing). The aim was to establish a baseline measurement for treatment effects. After 21 days of treatment, growth characteristics and biochemical data were collected on the plants subjected to the control and water stress treatments (Fig. 1).

### Morphological evaluation, relative water content, and water use efficiency

At the start of the treatments (baseline plants) and after 21 days (control and water stress treatments), the number of leaves, stem length, total fresh weight and fresh weight of each plant organ (leaf, stem and root) were determined. The roots of each plant, after cleaning and washing, were scanned and analysed using the RhizoVision Explorer software [25] to determine the area of the roots. Growth traits were analysed as the value of each plant under control or water-stressed conditions minus the average value of the baseline plants.

To measure the relative water content (RWC), a piece of about 1.5 × 1.5 cm was cut from the blade of the last fully expanded leaf and the fresh weight (FW) was taken, then it was hydrated in distilled water for 24 h to obtain the saturated weight (SW), and finally, it was placed in an oven at 75ºC for 72 h to obtain the dry weight (DW). From these data, the RWC was calculated as follows:

$$RWC=\frac{FW-DW}{SW-DW}$$

The water use efficiency (WUE) was determined from the total fresh weight (Total FW) and the fresh weight measured in the plants before the start of the treatments (Baseline FW) divided by the irrigation applied during the 21 days of treatment for each plant:

$$WUE=\frac{Total\,FW-Baseline\,FW}{Irrigation \left(ml\right)}$$

The day before the end of the experiment, after 20 days of treatment, the nitrogen balance index (NBI), which is a measure of the nitrogen status of the plant based on the ratio between chlorophyll and flavonoid content in plant leaves, was non-destructively measured using the Dualex® Scientific optical sensor (Force-A, Orsay, France) [26]. Data were collected from the adaxial and abaxial sides of two developed leaves from the top of the plant, and a mean was obtained for each experimental unit.

### Biochemical analyses

Biochemical analyses were carried out using spectrophotometry on different extracts from fresh leaves collected at the end of the experiment. To avoid dilution effects of the compounds by leaf water status, concentrations were expressed in dry weight (unit in weight of compound / DW of the leaf) by calculating the water content of a leaf sample from each plant.

To determine chlorophyll and carotenoid content in leaves, pigments were extracted from 0.1 g of leaf material in 1 mL of 80% (w/v) acetone. The samples were kept in the dark and mixed for 16 h before being centrifuged at 13,000 × *g* for 14 min at 4 °C to collect the supernatant. Using a spectrophotometer, the absorbance was measured at 470, 646 and 663 nm. Finally, pigment concentrations were calculated using the equations proposed by Lichtenthaler & Wellburn [27].

$$Chlorophyll\,a\,\left(\mu\,g\,{ml}^{-1}\right) ={(12.21A}_{663}-{2.81A}_{646})$$

$$Chlorophyll\,b\,\left(\mu\,g\,{ml}^{-1}\right) =(20.13{A}_{646}-{5.03A}_{663})$$

$$\begin{array}{l} Carotenoid\,\left(\mu\,g\,{ml}^{-1}\right)=\frac{{1000A}_{470}-3.27\left[Chlorophyll\,a\right]-104\left[Chlorophyll\,b\right]}{229}\end{array}$$

The hydrogen peroxide (H_2_O_2_) content was measured according to Loreto & Velikova [28]. Extraction was performed on 0.1 g of leaf material with 1 mL of 0.1% (w/v) trichloroacetic acid (TCA) solution. The extracts were centrifuged at 13,000 × g for 12 min at 4 °C, and 0.5 mL of the supernatant was mixed with 0.5 mL of 10 mM trisaminomethane at pH 7.0 and 1 mL of 1 M Kl. The absorbance was then measured at 510 nm, and the concentration was quantified using an H_2_O_2_ standard curve.

The concentrations of malondialdehyde (MDA), total phenolic compounds (TPC) and total flavonoids (TF) were determined from the same extract of 0.1 g of leaf material in 2 mL of 80% (w/v) methanol, mixed for 12 h and then centrifuged at 13,000 x *g* for 14 min at 4 °C to collect the supernatant. For MDA quantification, 0.2 mL of extracts were diluted in 0.4 mL of methanol and mixed with 0.6 mL of 0.5% (w/v) thiobarbituric acid (TBA) prepared in 20% (w/v) trichloroacetic acid (TCA), and for the blank of each sample, 0.2 mL of extracts were diluted in 0.4 mL of methanol and mixed with 0.6 mL of 20% TCA. The samples were incubated at 95 °C for 20 min, then placed on ice for 5 min to stop the reaction. The absorbance was then measured at 440, 532 and 600 nm, and the MDA concentration was calculated according to Hodges et al. [29].

Total phenolic compounds (TPC) were measured by reaction of the 0.1 mL of methanol extract diluted in 1.4 mL of H_2_O with 0.1 mL of Folin-Cicateau reagent [30], then adding 0.35 mL of 15% Na_2_CO_3_ 15% (w/v) and incubating at room temperature for 90 min in the dark. To quantify TPC, the absorbance was measured at 765 nm, and the concentration was calculated in relation to a standard curve with gallic acid. Meanwhile, TF quantification was performed according to Zhishen et al. [31]. First, 0.03 mL of 5% (w/v) NaNO_2_ was added to 0.05 mL of the methanol extract diluted in 0.45 mL of H_2_O, then 0.03 mL of 10% (w/v) AlCl_3_ and then 0.2 mL of 1 M NaOH. The absorbance was then measured at 510 nm, and the concentration was quantified using a catechin standard curve.

The proline content was determined according to the protocol of Bates et al. [32]. An extract was prepared from 0.1 g of leaf material in 1 mL of 3% sulphosalicylic acid. The extracts were centrifuged at 13,000 × *g* for 12 min at 4 °C, and 0.5 mL of the supernatant was mixed with 0.5 mL of ninhydrin acid and 0.5 mL of glacial acetic acid. The samples were incubated at 96 °C for 60 min, and then proline was extracted by adding 3 mL of toluene. Absorbance was measured at 520 nm, and the concentration was quantified using an L-proline standard curve.

### Statistical analysis

The experiment was conducted using a completely randomised design. Statistical analyses were performed using Infostat software version 2020 [33]. For each trait, data were analysed using two-way analysis of variance (ANOVA) with genotype and water treatment as the main factors. The statistical significance of the results was determined using a significance level of *p* < 0.05. Means were separated by the student-Newman-Keuls (SNK) multiple comparison test to determine differences between treatments and genotypes, using a significance level of *p* < 0.05.

To analyse the performance of the hybrids, the heterosis values over mid-parent (H_MP_) was calculated as [34, 35]:

$${H}_{MP}=100\times \frac{{F}_{1}-PM}{PM}$$

where F_1_ is the value of the hybrid and PM is the mean of the two parents. The significance of heterosis was determined by a Student’s *t* test, using a significance level of *p* < 0.05.

To evaluate the performance of the MAGIC lines, the percentage of increase or decrease was calculated with respect to the average of the MAGIC parents and with respect to the parent with the highest and lowest value for each trait. The significance of the percentage value was determined by a Student’s *t* test, using a significance level of *p* < 0.05.

Multivariate analysis was performed using R-studio [36]. Pairwise Pearson correlations were calculated between the analysed traits within each water treatment (control and water stress), and their significance was assessed at *p* < 0.001 with the Bonferroni correction using the R psych [37] and corrplot [38] packages. To identify relationships between the evaluated traits, genotypes and water treatments, a principal component analysis (PCA) was performed using the R package gglot2 [39].

## Results

### Analysis of variance

The two-way analysis of variance (ANOVA) revealed a significant (*p* < 0.05) effect of genotype, treatment, and genotype x treatment interaction for most of the traits evaluated, except that genotype had no significant effect on RWC, water treatment was non-significant for WUE and chlorophyll, and genotype x treatment interaction was non-significant for RWC, MDA and TF (Table 1). The genotype effect was the main contributor to the sum of squares (SS) for NBI, chlorophylls and carotenoids, whereas for irrigation amount, leaf number, stem length, total FW, leaf FW, stem FW, root FW, root area and proline the main contributor to SS was the treatment effect. The genotype x treatment effect was not the main contributor for any of the traits, and the residue had the highest percentage of SS for RWC, WUE, H_2_O_2_, MDA, TPC and TF (Table 1).

**Table 1:** ANOVA on eighteen eggplant genotypes under water stress and control (Treatment) and their interaction (Genotype x Treatment)

Trait	Genotype		Treatment		Genotype x Treatment		Residual
	SS (%)	*p*-value	SS (%)	*p*-value	SS (%)	*p*-value	SS (%)
Irrigation amount	11.6	< 0.0001	80.2	< 0.0001	2.9	< 0.0001	5.3
Leaf number	9.4	< 0.0022	48.1	< 0.0001	11.0	0.0004	31.6
Stem length	19.9	< 0.0001	44.0	< 0.0001	10.8	< 0.0001	25.3
Total FW	10.5	< 0.0001	59.5	< 0.0001	9.1	< 0.0001	21.0
Leaf FW	11.3	< 0.0001	61.5	< 0.0001	7.1	0.0003	20.1
Stem FW	21.0	< 0.0001	48.3	< 0.0001	8.9	< 0.0001	21.8
Root FW	29.2	< 0.0001	34.6	< 0.0001	10.4	< 0.0001	25.8
Root area	28.0	< 0.0001	30.6	< 0.0001	11.0	0.0002	30.3
RWC	12.2	0.1166	9.3	< 0.0001	9.5	0.3216	68.9
WUE	27.8	< 0.0001	1.3	0.0530	24.5	< 0.0001	46.4
NBI	41.0	< 0.0001	32.2	< 0.0001	8.5	< 0.0001	18.3
Chlorophylls	48.4	< 0.0001	0.7	0.2803	10.9	0.0232	40.1
Carotenoids	48.4	< 0.0001	2.2	0.0472	11.3	0.0145	38.2
H_2_O_2_	42.3	< 0.0001	1.4	0.0440	7.3	0.2563	49.0
MDA	17.3	0.0027	17.0	< 0.0001	6.4	0.5932	59.3
TPC	34.2	< 0.0001	2.7	0.0059	15.4	0.0009	47.8
TF	34.9	< 0.0001	7.9	< 0.0001	9.7	0.0523	47.5
Proline	6.3	0.0041	63.8	< 0.0001	7.6	0.0005	22.3

The numbers represent the percentage of the sum of squares (SS) and the *p*-value. The parameters evaluated were the amount of irrigation water (Irrigation amount), gains with respect to the baseline (value of control or water stress minus value of baseline plant) of leaf number, stem length, total fresh weight (Total FW), leaf fresh weight (Leaf FW), stem fresh weight (Stem FW), root fresh weight (Root FW) and root area, and relative water content (RWC), water use efficiency (WUE), nitrogen balance index (NBI), total chlorophylls, total carotenoids, hydrogen peroxide (H_2_O_2_), malondialdehyde (MDA), total phenolic compounds (TPC), total flavonoids (TF) and proline

### Growth and biochemical responses of the eight parents of the MAGIC population

**Fig. 2:**
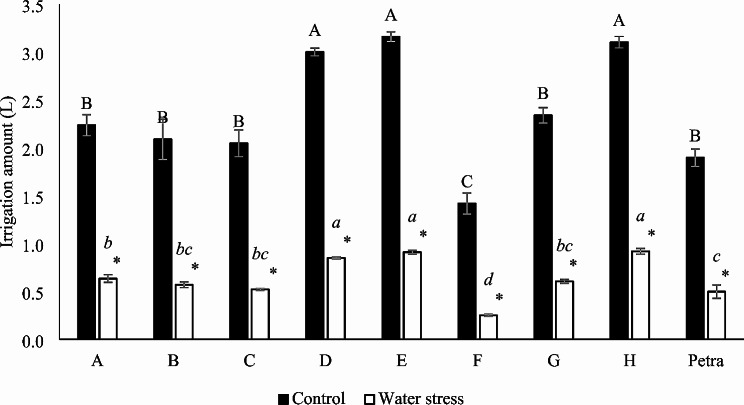
Irrigation applied during the 21 days of water treatment of the eight parents of the MAGIC line and the commercial hybrid Petra under both control (100% FC) and water stress (30% FC) conditions. Different letters in each treatment (uppercase letter for control and lowercase italics for water stress) indicate significant differences between genotypes, determined using the SNK multiple comparison test at a significance level of *p* < 0.05. The asterisk indicates a significant difference between the control and water stress treatments for each genotype, according to the SNK method for a *p*-value < 0.05. Vertical bars indicate ± standard error

The irrigation amount for eight parents of the MAGIC population and the commercial hybrid Petra averaged 2.37 L and 0.64 L during the 21 days of treatment for the control (100% FC) and water stress (30% FC) treatments, respectively. The nine genotypes displayed similar patterns regarding the consumption of water in both irrigation conditions, with D, E and H consuming high amounts of water, A, B, C, G and Petra having an intermediate water consumption and F having a lower consumption of the available water (Fig. 2).

Water stress caused a significant decrease in the number of leaves in all genotypes except for B (Table 2). Comparing the selected genotypes, there were no significant differences within the control treatment. In contrast, under water stress, genotype F had a higher number of leaves than genotypes D, E and H. Stem length showed less growth under water stress conditions in six parents and Petra, whereas genotypes B and F had similar growth under both irrigation conditions. The genotypes with the highest stem length growth under both irrigation conditions were D, E and H; the stem did not lengthen in genotypes C and Petra during the 21 days of reduced irrigation (Table 2).

**Table 2:** Differences in the values of growth and physiological traits under control and water stress conditions

Trait	Treatment	A	B	C	D	E	F	G	H	Petra
Leaf number	Control	1.8 a	1.8 a	2.2 a	3.8 a	3.2 a	0.8 a	1.4 a	3.1 a	2.2 a
	Water stress	-0.2 *ab*	0.2 *ab*	-0.6 *ab*	-0.8 *b*	-1.0 *b*	1.6 *a*	-0.4 *ab*	-1.6 *b*	0.6 *ab*
	*Change %*	*-111* ^**^	*-89* ^*ns*^	*-127* ^**^	*-121* ^***^	*-131* ^***^	*100* ^*ns*^	*-129* ^*^	*-152* ^***^	*-73* ^*^
Stem length (cm)	Control	6.5 bc	4.5 cd	3.4 cd	8.9 ab	8.1 ab	1.8 d	4.3 cd	10.4 a	3.0 cd
	Water stress	1.4 *abc*	2.4 *a*	0.0 *c*	2.0 *ab*	0.3 *abc*	1.4 *abc*	0.7*abc*	2.1 *ab*	0.0 *bc*
	*Change %*	*-78* ^***^	*-47* ^*ns*^	*-100* ^**^	*-78* ^***^	*-96* ^***^	*-23* ^*ns*^	*-84* ^**^	*-80* ^***^	*-101* ^*^
Total FW (g)	Control	31.6 a	24.8 ab	12.5 bc	36.9 a	36.9 a	9.6 c	29.2 a	38.3 a	21.0 abc
	Water stress	10.0 *a*	9.0 *ab*	3.3 *b*	6.4 *ab*	3.9 *ab*	4.8 *ab*	6.7 *ab*	6.8 *ab*	7.9 *ab*
	*Change %*	*-68* ^**^	*-64* ^*ns*^	*-74* ^***^	*-83* ^***^	*-89* ^***^	*-50* ^*ns*^	*-77* ^***^	*-82* ^***^	*-63* ^*^
Leaf FW (g)	Control	18.9 a	14.5 a	5.0 b	15.0 a	11.1 ab	4.7 b	15.1 a	15.3 a	13.9 a
	Water stress	5.2 *a*	5.0 *a*	0.4 *b*	-0.1 *b*	-3.8 *c*	2.7 *ab*	2.2 *ab*	0.1 *b*	4.7 *a*
	*Change %*	*-72* ^***^	*-65* ^*^	*-92* ^***^	*-101* ^***^	*-134* ^***^	*-43* ^*ns*^	*-85* ^***^	*-99* ^***^	*-66* ^*^
Stem FW (g)	Control	4.3 bcd	3.2 cd	1.8 d	5.4 bc	6.2 b	2.8 cd	4.6 bcd	8.7 a	2.8 cd
	Water stress	1.1 *bcd*	1.2 *bcd*	0.8 *cd*	1.7 *bc*	1.8 *b*	0.6 *d*	1.2 *bcd*	2.6 *a*	1.1 *bcd*
	*Change %*	*-73* ^**^	*-61* ^*ns*^	*-56* ^**^	*-69* ^***^	*-71* ^***^	*-78* ^*ns*^	*-73* ^***^	*-70* ^***^	*-61* ^**^
Root FW (g)	Control	8.6 cd	7.2 cd	6.1 cd	17.5 a	21.0 a	2.2 d	9.9 bc	15.0 ab	4.5 cd
	Water stress	3.9 *bcd*	2.9 *cd*	2.6 *d*	5.9 *ab*	7.2 *a*	1.6 *d*	3.7 *bcd*	5.0 *bc*	2.3 *d*
	*Change %*	*-55* ^*^	*-60* ^*ns*^	*-58* ^***^	*-67* ^**^	*-66* ^***^	*-26* ^*ns*^	*-63* ^**^	*-66* ^***^	*-50* ^*ns*^
Root area (cm^2^)	Control	746.8 bcd	499.5 cde	431.0 de	1204.4 ab	1328.4 a	169.0 e	763.6 bcd	1015.9 abc	356.4 de
	Water stress	371.6 *bc*	279.5 *bc*	202.7 *c*	489.0 *ab*	588.1 *a*	184.2 *c*	355.5 *bc*	390.0 *bc*	198.2 *c*
	*Change %*	*-50* ^*^	*-44* ^*ns*^	*-53* ^**^	*-59* ^**^	*-56* ^**^	*9* ^*ns*^	*-53* ^*^	*-62* ^***^	*-44* ^*ns*^
RWC (%)	Control	83.1 a	80.3 a	72.6 a	77.1 a	69.3 a	81.8 a	80.1 a	79.1 a	82.0 a
	Water stress	73.3 *a*	79.7 *a*	69.1 *a*	70.5 *a*	70.9 *a*	62.5 *a*	68.2 *a*	67.1 *a*	75.2 *a*
	*Change %*	*-12* ^*^	*-1* ^*ns*^	*-5* ^*ns*^	*-9* ^*ns*^	*2* ^*ns*^	*-24* ^*ns*^	*-15* ^*^	*-15* ^**^	*-8* ^*ns*^
WUE	Control	13.9 a	10.9 ab	6.2 b	12.3 ab	11.6 ab	6.4 b	12.5 ab	11.3 ab	10.9 ab
	Water stress	15.7 *ab*	15.5 *ab*	6.3 *cd*	7.5 *bcd*	4.3 *d*	18.4 *a*	11.1 *abcd*	7.3 *bcd*	14.4 *abc*
	*Change %*	*13* ^*ns*^	*42* ^*ns*^	*2* ^*ns*^	*-39* ^**^	*-63* ^**^	*186* ^*^	*-11* ^*ns*^	*-36* ^*ns*^	*32* ^*ns*^
NBI	Control	21.0 ab	19.4 bc	24.8 a	12.3 d	10.6 d	21.7 ab	23.4 ab	16.5 c	19.5 bc
	Water stress	24.3 *bc*	25.3 *ab*	21.6 *cd*	18.3 *e*	16.8 *e*	25.8 *ab*	28.2 *a*	20.0 *de*	24.5 *bc*
	*Change %*	*16* ^*ns*^	*30* ^*^	*-13* ^*^	*49* ^**^	*58* ^***^	*19* ^*ns*^	*20* ^***^	*21* ^*ns*^	*26* ^***^

Traits evaluated: Leaf number, stem length, total fresh weight (Total FW), leaf fresh weight (Leaf FW), stem fresh weight (Stem FW), root fresh weight (Root FW) and root area for control (100% FC) and water stress (30% FC) conditions, expressed as gains over the baseline measurements (value of control or water stress condition minus value of baseline plant), and relative water content (RWC), water use efficiency (WUE) and nitrogen balance index (NBI) for the eight parents of the MAGIC line and the commercial F_1_ hybrid Petra. Change (%) represents the percentage change of the water stress value relative to the control value. Different letters within each row indicate significant differences between genotypes, according to SNK method at a *p*-value < 0.05. ^ns^, ^*^, ^**^, ^***^ indicate, respectively, non-significant at a *p*-value < 0.05 and significant for a *p*-value < 0.05, < 0.01 and < 0.001 for the difference between the control and water stress treatment, for each genotype

Total FW gain was affected by water stress with reductions up to 89% (genotype E), but genotypes B and F were not significantly affected by treatments. Genotypes with high growth in both treatments were A, B, D, E, G, H and Petra, with F being the genotype with the lowest mean growth under control conditions (9.6 g) and C being the genotype with the lowest mean growth under water stress (3.3 g) (Table 2). The reduction in total FW gain under water stress was due to the reduction in the three plant organs, with the leaf, stem and root showing an average reduction in growth of 85.6, 69.4 and 62.8%, respectively. The genotypes with the highest gain in leaf FW in both conditions were A, B, F, G and Petra, whereas genotypes D and E even showed negative growth under stress (due to leaf abscission). For stem FW gain, only genotype H stood out in both growth conditions; for root FW gain, genotypes D and E showed the highest growth in both conditions. Root area decreased on average by 45.0% when grown under water stress, with genotypes D and E showing superior growth in both conditions.

Compared to control conditions, RWC decreased significantly under water stress for A, G and H, but the irrigation treatment had no significant effect on the other six genotypes; no genotype was significantly different from the others within each treatment (Table 2). WUE decreased under water stress conditions with respect to the control in D and E, but increased by 186% in F. Regarding genotype effects, A, B, G and H showed higher values than the other genotypes in both treatments (Table 2). As for NBI, water stress caused an increase over the control in five genotypes (B, D, E, G, Petra), but a decrease in C. The genotypes with the highest values for NBI in both environments were F and G (Table 2).

**Fig. 3:**
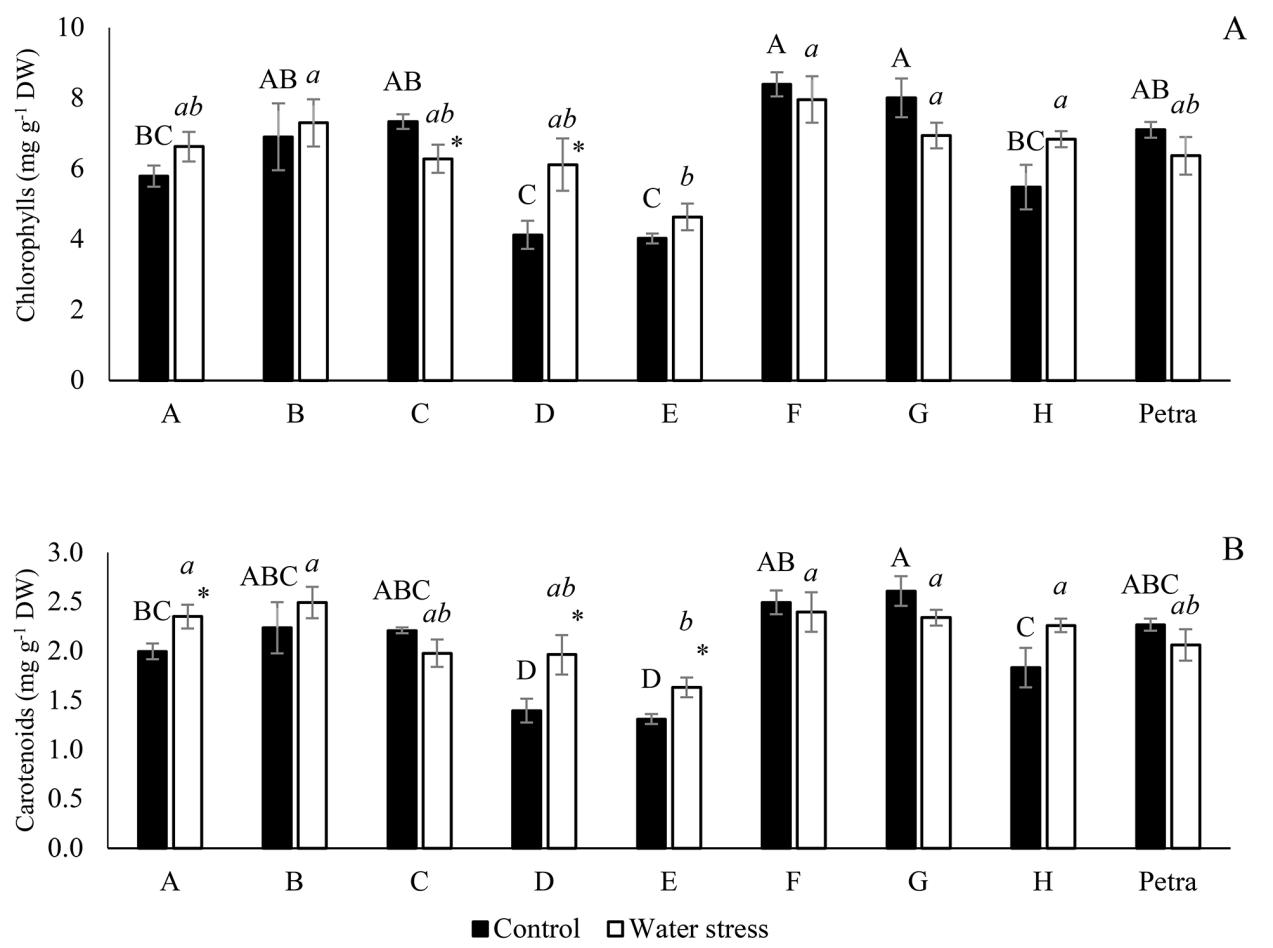
Chlorophyll a (**A**), chlorophyll b (**B**), total chlorophyll (**C**) and carotenoids (**D**) of the eight parents of the MAGIC line and the commercial hybrid Petra under control (100% FC) and water stress (30% FC) conditions. Different letters in each treatment (uppercase letter for control and lowercase italics for water stress) indicate significant differences between genotypes, determined using the SNK multiple comparison test at a significance level of *p*-value < 0.05. The asterisk indicates a significant difference between the control and water stress treatments for each genotype, according to the SNK method for a *p*-value < 0.05. Vertical bars indicate ± standard error

Regarding the photosynthetic pigments, the treatments did not affect most of the genotypes (Fig. 3). Chlorophylls contents decreased in C and increased in D under water stress. Carotenoids increased under water stress conditions in genotypes A, D and E. On the other hand, the genotypes having higher values in both conditions were B, C, F, G and Petra for both chlorophylls and carotenoids; genotype E was the one that had the lowest average value in both conditions (Fig. 3).

As for the oxidative stress parameters, H_2_O_2_ increased by 182% in G when grown under water stress, but the rest of the genotypes were unaffected. The genotypes with the highest H_2_O_2_ levels in both treatments were A, F, G and H (Table 3). The MDA content increased under stress conditions in genotypes B, C, D and G. Within the control treatment, the MDA content was higher in F than in D, whereas under water stress, there were no differences between the genotypes. For the non-enzymatic antioxidants, TPC increased in B and C and decreased in A and D, and TF decreased in A but did not change significantly in the other genotypes (Table 3). The genotypes with higher TPC content in both treatments were A, E, G and H, whereas C was the only genotype that showed lower TF, being significantly lower than most of the other genotypes (Table 3).

**Table 3:** Differences in the values of oxidative stress markers and antioxidant compounds

Trait		A	B	C	D	E	F	G	H	Petra
H_2_O_2_ (µmoles g^− 1^ DW)	Control	8.5 a	2.7 a	2.2 a	3.5 a	3.9 a	6.7 a	2.5 a	6.9 a	2.6 a
	Water stress	8.9 *a*	3.1 b	2.2 b	3.8 b	3.5 b	9.5 a	7.0 *ab*	5.3 *ab*	2.9 *b*
	*Change %*	*4*	*15*	*1*	*8*	*-11*	*42*	*182* ^*^	*-23*	*11*
MDA (µmoles g^− 1^ DW)	Control	315.5 ab	270.7 ab	381.5 ab	139.0 b	233.3 ab	406.4 a	295.1 ab	235.3 ab	272.3 ab
	Water stress	373.5 *a*	448.2 *a*	598.6 *a*	426.8 *a*	352.0 *a*	527.7 *a*	504.1 *a*	346.7 *a*	334.8 *a*
	*Change %*	*18*	*66* ^*^	*57* ^*^	*207* ^***^	*51*	*30*	*71* ^*^	*47*	*23*
TPC (mg eq. GA g^− 1^ DW)	Control	35.1 ab	14.8 d	13.6 d	27.7 abc	30.0 ab	23.7 bcd	33.6 ab	37.0 a	18.9 cd
	Water stress	28.1 ab	30.8 *ab*	16.8 *c*	20.8 *bc*	35.3 *a*	17.4 *c*	29.9 *ab*	30.3 *ab*	21.7 *bc*
	*Change %*	*-20* ^**^	*108* ^*^	*23* ^*^	*-25*	*18*	*-26* ^*^	*-11*	*-18*	*15*
TF (mg eq. C g^− 1^ DW)	Control	15.7 a	7.0 ab	4.0 b	12.5 a	14.2 a	9.1 ab	15.5 a	16.3 a	7.0 ab
	Water stress	9.9 ab	11.7 *a*	3.6 *b*	6.6 *ab*	14.1 *a*	6.2 *ab*	13.9 *a*	12.7 *a*	8.3 *ab*
	*Change %*	*-37* ^*^	*66*	*-9*	*-47*	*-1*	*-32*	*-10*	*-22*	*19*

Traits evaluated: Hydrogen peroxide (H_2_O_2_), malondialdehyde (MDA), total phenolic compounds (TPC) and total flavonoids (TF), for control (100% FC) and water stress (30% FC) conditions, for the eight parents of the MAGIC line and the commercial F_1_ hybrid Petra. Change represents the percentage change of the water stress value relative to the control value. Different letters within each row indicate significant differences between genotypes, according to SNK method at a *p*-value < 0.05. ^ns^, ^*^, ^**^, ^***^ indicate, respectively, non-significant at a *p*-value < 0.05 and significant for a *p*-value < 0.05, < 0.01 and < 0.001 for the difference between the control and water stress treatment, for each genotype.

**Fig. 4:**
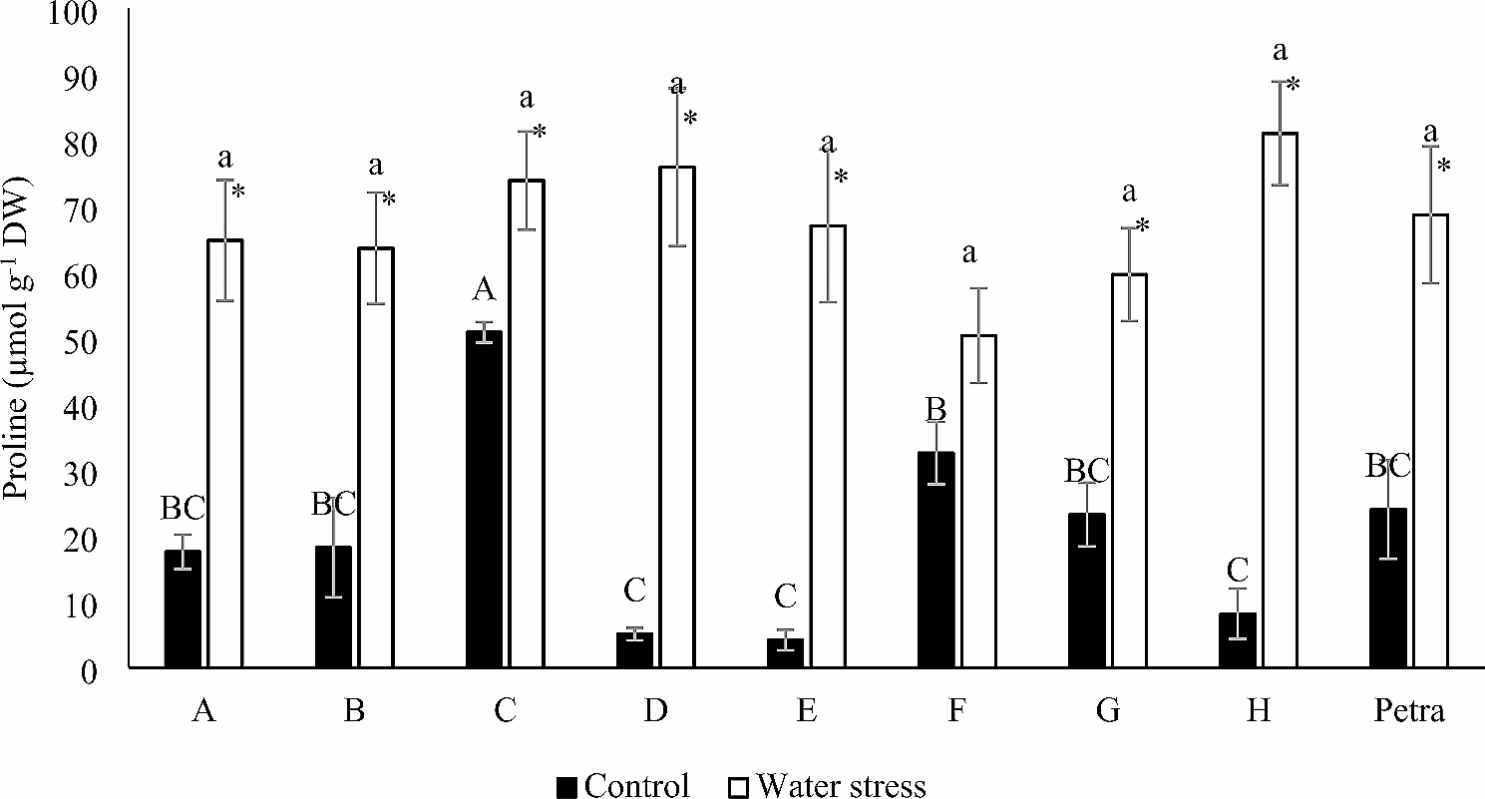
Proline of the eight parents of the MAGIC line and the commercial hybrid Petra under control and water stress conditions. Different letters in each treatment (uppercase letter for control and lowercase italics for water stress) indicate significant differences between genotypes, determined using the SNK multiple comparison test at a significance level of *p* < 0.05. The asterisk indicates a significant difference between the control and water stress treatments for each genotype, according to the SNK method for a *p*-value < 0.05. Vertical bars indicate ± standard error

Proline content increased significantly in all genotypes, except for F, under the water stress treatment. Increases of proline in the other genotypes ranged from 45.2% in genotype C to 1495.0% in genotype E. Under control conditions, genotype C showed a higher proline content, whereas, under water stress conditions, no genotype showed values significantly different than the others (Fig. 4).

### Response of F_1_ hybrids and MAGIC lines

The four hybrids evaluated, like their parents, were affected in their growth under water stress conditions. To evaluate their performance in relation to the parents, heterosis was calculated for each parameter evaluated (Table 4). In this way, hybrid A × B showed heterotic effects with a higher water consumption (irrigation) under water stress and a higher TPC content under control conditions, but negative heterosis effects were also detected for total FW, leaf FW, RWC, WUE, carotenoids and proline under water stress and also H_2_O_2_ under both irrigation conditions. The hybrid C × D showed no significant heterotic effects with respect to the parents, except for NBI, which had positive heterosis under water stress and for proline, which had negative heterosis under water stress. Similarly, hybrid E × F showed significant heterotic effects in only two parameters: positive heterosis for leaf FW under control conditions but negative heterosis under water stress, whereas NBI displayed positive heterosis under water stress. Finally, hybrid G × H showed significant positive heterotic effects for irrigation amount, leaf number, root FW under water stress conditions and significant negative heterosis for stem length, stem FW, WUE, H_2_O_2_ and proline under water stress, NBI in both irrigation conditions, and chlorophylls and carotenoids in control conditions (Table 4).

**Table 4:** Analysis of the four F_1_ hybrids with respect to their parents

Trait	Treatment	A × B			C × D			E × F			G × H		
		F_1_	Pm	H_MP_	F_1_	Pm	H_MP_	F_1_	Pm	H_MP_	F_1_	Pm	H_MP_
Irrigation amount (L)	Control	2.7	2.2	23.4	2.4	2.5	-4.4	2.7	2.3	16.8	3.1	2.7	12.9
	Water stress	0.9	0.6	49.8^*^	0.5	0.7	-24.4	0.6	0.6	9.0	0.9	0.8	23.5^*^
Leaf number	Control	0.8	1.8	-55.6	2.0	3.0	-34.2	2.6	2.0	30.0	2.0	2.2	-10.7
	Water stress	-1.6	0.0	∞	0.5	-0.7	-171.4	0.0	0.3	-100.0	-1.8	-1.0	80.0^*^
Stem length (cm)	Control	4.0	5.5	-27.2	3.6	6.1	-40.9	7.6	4.9	54.4	5.3	7.4	-28.5
	Water stress	1.6	1.9	-17.3	1.0	0.8	26.6	0.9	0.9	3.5	0.1	1.4	-95.7^*^
Total FW (g)	Control	21.6	28.2	-23.2	23.0	24.7	-7.0	37.0	23.3	59.1	36.4	33.8	7.9
	Water stress	3.2	9.5	-66.0^*^	6.1	4.8	25.3	7.9	4.4	81.3	4.5	6.8	-33.3
Leaf FW (g)	Control	10.2	16.7	-38.8	10.5	10.0	5.0	18.2	7.9	129.7^*^	15.2	15.2	-0.3
	Water stress	-0.7	5.1	-114.4^*^	2.4	0.1	1800.0	4.2	-0.5	-903.1^*^	-0.7	1.2	-160.9
Stem FW (g)	Control	4.3	3.8	15.2	3.1	3.6	-14.6	5.3	4.5	17.7	5.0	6.7	-25.1
	Water stress	1.4	1.2	19.8	0.8	1.2	-38.4	1.1	1.2	-12.2	1.3	1.9	-33.3^*^
Root FW (g)	Control	8.5	7.9	8.1	9.9	11.8	-16.3	13.9	11.6	20.1	18.0	12.5	44.1
	Water stress	4.0	3.4	19.3	3.3	4.2	-21.9	3.1	4.4	-30.9	5.7	4.4	29.2^*^
Root area (cm^3^)	Control	565.2	623.1	-9.3	640.1	817.7	-21.7	1149.2	748.7	53.5	1205.3	889.8	35.5
	Water stress	430.7	325.5	32.3	271.6	345.9	-21.5	296.1	386.1	-23.3	408.2	372.7	9.5
RWC (%)	Control	73.5	81.7	-10.1	73.1	74.8	-2.3	83.2	75.5	10.2	66.1	79.6	-17.0
	Water stress	69.4	76.5	-9.3^*^	77.4	69.8	11.0	64.6	66.7	-3.2	68.4	67.6	1.2
WUE	Control	7.2	12.4	-42.0	10.1	9.3	8.6	12.9	9.0	42.7	11.7	11.9	-1.8
	Water stress	3.5	15.6	-77.9^*^	11.3	6.9	63.2	11.4	11.4	0.7	4.7	9.2	-49.3^*^
NBI	Control	15.2	20.2	-24.8	17.88	18.6	-3.7	17.5	16.2	8.5	15.5	20.0	-22.2^*^
	Water stress	23.3	24.8	-5.9	23.82	19.9	19.5^*^	27.6	21.3	29.5^*^	22.0	24.1	-8.6^*^
Chlorophylls (mg g^− 1^ DW)	Control	4.8	6.3	-24.9	6.2	5.7	9.1	5.6	6.2	-9.4	4.6	6.7	-31.3^*^
	Water stress	6.0	7.0	-13.6	8.9	6.2	44.1	6.7	6.3	6.7	6.4	6.9	-7.2
Carotenoids (mg g^− 1^ DW)	Control	1.5	2.1	-28.5	1.9	1.8	6.8	1.7	1.9	-11.9	1.5	2.2	-32.0^*^
	Water stress	2.0	2.4	-16.5^*^	2.8	2.0	41.9	2.2	2.0	8.8	2.3	2.3	-1.8
H_2_O_2_ (µmoles g^− 1^ DW)	Control	3.5	5.6	-37.1^*^	1.5	2.9	-47.7	5.5	5.3	3.4	3.1	4.7	-34.3
	Water stress	3.8	6.0	-36.2^*^	2.7	3.0	-10.6	5.9	6.5	-8.8	1.5	6.2	-75.0^*^
MDA (µmoles g^− 1^ DW)	Control	277.5	293.1	-5.3	332.5	260.3	27.8	270.8	319.8	-15.3	189.4	265.2	-28.6
	Water stress	376.6	410.8	-8.3	433.4	512.7	-15.5	461.6	439.9	4.9	317.8	425.4	-25.3
TPC (mg eq GA g^− 1^ DW)	Control	33.6	25.5	31.4^*^	27.4	20.7	32.5	28.3	26.9	5.2	26.1	35.3	-26.1
	Water stress	27.2	27.5	-1.3	20.8	18.8	10.5	26.3	26.6	-1.2	30.7	30.1	1.8
TF (mg eq C g^− 1^ DW)	Control	15.4	11.3	35.9	11.2	8.3	35.3	12.7	11.6	8.8	16.8	15.9	5.5
	Water stress	12.4	10.8	14.3	6.9	5.1	34.5	8.2	10.1	-18.8	13.3	13.3	0.0
Proline (µmol g^− 1^ DW)	Control	10.8	17.9	-39.6^*^	34.9	28.0	24.6	16.2	18.4	-11.9	7.8	15.7	-50.1^*^
	Water stress	62.1	64.2	-3.3	49.0	74.9	-34.6^*^	74.5	58.7	27.0	57.8	70.3	-17.8

F_1_ is the mean value of the hybrid, Pm is the mean value of the parents, and H_MP_ is the hybrid mid parental heterosis. For the traits related to plant growth or biomass, they are expressed as gains over baseline measurements (value of control or water stress condition minus value of baseline plant). The asterisk indicates a significant difference between F_1_ and Pm, according to the test *t* for a *p*-value < 0.05

To compare the five MAGIC S5 lines with their parents, the percentage increase or decrease was calculated with respect to the mean of the parents and with the highest and lowest parental values for each trait evaluated in each treatment. It can be seen that no line was inferior to the lower parent or superior to the higher parent for any of the traits, with the exception of TPC, which increased by 20.8% in line M45 under control conditions (Table 5). Line M40 showed higher leaf number, RWC and TPC content, and lower irrigation, stem length, stem FW, H_2_O_2_ and proline content than the mean of the parents under control conditions; under water stress, it had higher values for leaf number, leaf FW and NBI, and lower values for H_2_O_2_ and proline. Regarding line M45, it showed higher growth than the average of the parents, with higher values of irrigation, stem length, total FW, stem FW, root FW and TF, and lower values of NBI, H_2_O_2_ MDA in both treatments, as well as higher values for the number of leaves, root area and TPC, and lower values of chlorophyll and carotenoids in control conditions. In the case of line M194, it had higher values than the average of the parents for irrigation in control conditions, and for irrigation, WUE and NBI in the water stress condition, but lower values for H_2_O_2_ and proline in control conditions and for leaf FW, H_2_O_2_ and TF under water stress. Line M204 had higher values than the average of the parents for the traits RWC and MDA in control and for stem length, stem FW, RWC, NBI, H_2_O_2_ and TF in water stress and lower values than the average of the parents for the traits irrigation, root FW, root area in control and root FW, root area in water stress. Finally, line M262 displayed higher values than the parental averages for the traits stem length, total FW, leaf FW, stem FW, TPC and TF in control conditions, and H_2_O_2_ in water stress conditions, whereas it showed lower values for the traits WUE, NBI, chlorophylls, carotenoids and proline in control conditions and leaf number and root FW in water stress conditions (Table 5).

**Table 5:** Percentage increase or decrease of the MAGIC S5 lines compared to the eight parents in control (C) and water stress (WS) conditions

Trait	Parents	M40		M45		M194		M204		M262	
		C	WS	C	WS	C	WS	C	WS	C	WS
Irrigation amount	Mean	-18.8^*^	-1.7	25.5^*^	29.9^*^	25.4^*^	22.4^*^	-17.3^*^	-14.1	4.6	-12.7
	Highest	-37.7^*^	-29.7^*^	-3.8^*^	-7.0	-3.9	-12.4	-36.5^*^	-38.5^*^	-19.8^*^	-37.5^*^
	Lowest	38.8^*^	156.7^*^	114.5^*^	239.4^*^	114.2^*^	219.7^*^	41.4^*^	124.3^*^	78.8^*^	128.1^*^
Leaf number	Mean	59.8^*^	427.3^*^	24.3^*^	-145.5	15.4	18.2	-37.9	-145.5	-20.1	-309.1^*^
	Highest	-5.3	-50.0^*^	-26.3^*^	-137.5^*^	-31.6^*^	-112.5^*^	-63.2^*^	-137.5^*^	-52.6^*^	-162.5^*^
	Lowest	350.0^*^	150.0^*^	250.0^*^	62.5^*^	225.0^*^	87.5^*^	75.0	62.5	125.0^*^	37.5
Stem length	Mean	-37.6^*^	-10.2	89.5^*^	98.0^*^	26.8	55.8	-0.1	125.5^*^	83.9^*^	118.1
	Highest	-66.2^*^	-59.2^*^	2.6	-10.0	-31.3^*^	-29.2	-45.9^*^	2.5	-0.4	-0.8
	Lowest	99.7^*^	313.0^*^	507.1^*^	569.6^*^	306.2^*^	469.6^*^	220.0^*^	634.8^*^	488.9^*^	617.4^*^
Total FW	Mean	-15.5	28.4	36.6^*^	23.9^*^	23.0	-50.6	-27.0	4.8	24.3^*^	-23.5
	Highest	-41.0^*^	-16.2	-4.6	-19.1^*^	-14.0	-67.7^*^	-49.0^*^	-31.6	-13.2	-50.0^*^
	Lowest	135.0^*^	156.3^*^	280.0^*^	147.2^*^	242.3^*^	-1.3	103.2	109.2^*^	245.9^*^	52.7
Leaf FW	Mean	9.0	130.8^*^	24.4	-21.9	25.8	-130.1^*^	-17.5	33.3	40.8^*^	-1.6
	Highest	-27.3	-19.4	-17.0	-72.7^*^	-16.0	-110.5^*^	-44.9^*^	-53.4^*^	-6.1	-65.6^*^
	Lowest	191.4^*^	212.7^*^	232.8^*^	138.1^*^	236.5^*^	85.3^*^	120.8^*^	165.1^*^	276.5^*^	148.1^*^
Stem FW	Mean	-44.1^*^	-18.6	47.6^*^	37.9^*^	7.6	16.8	-10.5	54.1^*^	53.3^*^	10.6
	Highest	-71.5^*^	-57.4^*^	-24.8^*^	-27.9^*^	-45.2^*^	-38.9	-54.4^*^	-19.4	-21.9^*^	-42.2^*^
	Lowest	34.5	76.9^*^	255.4^*^	199.7^*^	159.0^*^	153.8	115.5^*^	234.9^*^	269.0^*^	140.4^*^
Root FW	Mean	-32.5	-7.7	47.4^*^	43.6^*^	25.1	-29.7	-46.0^*^	-30.8^*^	-9.6	-42.0^*^
	Highest	-67.1^*^	-50.2^*^	-28.1^*^	-22.5^*^	-39.0^*^	-62.1^*^	-73.7^*^	-62.6^*^	-55.9^*^	-68.7^*^
	Lowest	214.1^*^	120.9^*^	586.2^*^	243.6^*^	482.6^*^	68.2	151.5	65.6	320.9^*^	38.8
Root area	Mean	-33.4	-8.5	45.3^*^	18.9	15.2	-23.5	-44.3^*^	-23.3^*^	-1.1	-39.6
	Highest	-61.4^*^	-44.4^*^	-15.8^*^	-27.7^*^	-33.2^*^	-53.5^*^	-67.7^*^	-53.4^*^	-42.7^*^	-63.3^*^
	Lowest	203.3^*^	77.5	561.6^*^	130.8^*^	424.9^*^	48.4	153.5	48.8^*^	350.6^*^	17.2
RWC	Mean	9.7^*^	0.9	-7.9	2.7	-1.9	-5.2	7.4^*^	13.9^*^	-1.7	4.8
	Highest	2.9	-11.1	-13.6	-9.6	-8.0	-16.5^*^	0.7	0.3	-7.9	-7.7
	Lowest	23.4^*^	13.3	3.6	15.3^*^	10.3	6.4	20.8^*^	27.9^*^	10.5	17.7^*^
WUE	Mean	5.0	11.1	-7.6	21.7	4.4	1.0^*^	-6.3	-5.6	-2.0^*^	7.6
	Highest	-17.3	-10.5	-27.2	-2.0^*^	-17.7^*^	-18.7^*^	-26.1^*^	-24.0	-22.8	-13.3
	Lowest	60.9^*^	48.3^*^	41.6^*^	62.5^*^	60.0^*^	34.8	43.6	26.0^*^	50.2^*^	43.7
NBI	Mean	20.0	29.4^*^	-20.2^*^	-8.3^*^	-6.8	13.2^*^	6.3	15.5^*^	-24.7^*^	10.2
	Highest	-9.3	3.4	-39.8^*^	-26.7^*^	-29.6^*^	-9.6^*^	-19.7^*^	-7.7^*^	-43.2^*^	-11.9^*^
	Lowest	111.3^*^	73.5^*^	40.4^*^	22.9^*^	64.1^*^	51.8^*^	87.2^*^	54.9^*^	32.5^*^	47.8^*^
Chlorophyll	Mean	16.1	33.4	-30.9^*^	-17.4	-19.5	0.9	15.3	9.0	-22.3^*^	14.6
	Highest	-13.4	10.4	-48.5^*^	-31.7^*^	-40.0^*^	-16.5	-14.1	-9.8	-42.0^*^	-5.1
	Lowest	80.4^*^	89.5^*^	7.3	17.3	25.1	43.3^*^	79.1^*^	54.9^*^	20.8	62.9^*^
Carotenoids	Mean	20.0	33.8	-32.9^*^	-16.2	-21.2	6.8	21.0	14.8	-22.0^*^	12.1
	Highest	-7.5	16.8	-48.3^*^	-26.8^*^	-39.3^*^	-6.7	-6.7	0.2	-39.8^*^	-2.1
	Lowest	84.2^*^	78.5^*^	3.0	11.8	20.9	42.5^*^	85.7^*^	53.1^*^	19.8	49.6^*^
H_2_O_2_	Mean	-67.5^*^	-64.1^*^	-54.5^*^	-58.9^*^	-51.9^*^	-52.7^*^	76.0	57.2^*^	18.3	135.6^*^
	Highest	-82.4^*^	-79.5^*^	-75.3^*^	-76.5^*^	-73.9^*^	-72.9^*^	-4.5	-10.1	-35.8^*^	34.7
	Lowest	-31.8	-12.6	-4.5	0.0	0.9	15.3	269.1^*^	282.9^*^	148.2^*^	473.9^*^
MDA	Mean	47.1	-16.6	-12.7^*^	-28.5^*^	-23.6	-11.3	40.6^*^	-5.4	-20.7	-8.5
	Highest	3.0	-37.7^*^	-38.9^*^	-46.6^*^	-46.5^*^	-33.8^*^	-1.6	-29.3^*^	-44.5^*^	-31.7^*^
	Lowest	201.1^*^	7.5	78.6^*^	-7.8	56.3	14.3	187.7^*^	22.1	62.3	17.9
TPC	Mean	22.6^*^	5.8	38.7^*^	-3.4	7.4	-26.2	16.9	14.3	37.7^*^	0.4
	Highest	-10.8^*^	-21.4^*^	0.9	-28.3^*^	-21.8^*^	-45.2^*^	-14.9	-15.1	0.3	-25.5^*^
	Lowest	142.0^*^	65.4^*^	173.8^*^	51.0	112.0^*^	15.4	130.8^*^	78.7^*^	172.0^*^	56.8^*^
TF	Mean	17.9	13.3	67.1^*^	12.5^*^	13.2	-47.2^*^	6.0	33.4^*^	42.2^*^	10.0
	Highest	-14.8	-21.1^*^	20.8^*^	-21.6^*^	-18.2	-63.3^*^	-23.3	-7.1	2.8	-23.4
	Lowest	248.7^*^	207.8^*^	394.2^*^	205.7^*^	234.8^*^	43.3	213.6^*^	262.4^*^	320.6^*^	198.9^*^
Proline	Mean	-59.6^*^	-33.8^*^	-13.5	-22.0	-72.5^*^	-0.8	25.8	0.4	-68.7^*^	6.4
	Highest	-84.1^*^	-45.2^*^	-66.0^*^	-35.5^*^	-89.2^*^	-17.9	-50.6^*^	-17.0	-87.7^*^	-12.0
	Lowest	92.4	-11.9	311.7^*^	3.8	30.8	32.0	498.5^*^	33.6	48.9	41.5

The values of the lines were compared with the mean, highest and lowest values in the eight MAGIC parents. The asterisk indicates a significant difference between MAGIC line and parents, according to the test *t* for a *p*-value < 0.05

### Multivariate analysis

**Fig. 5:**
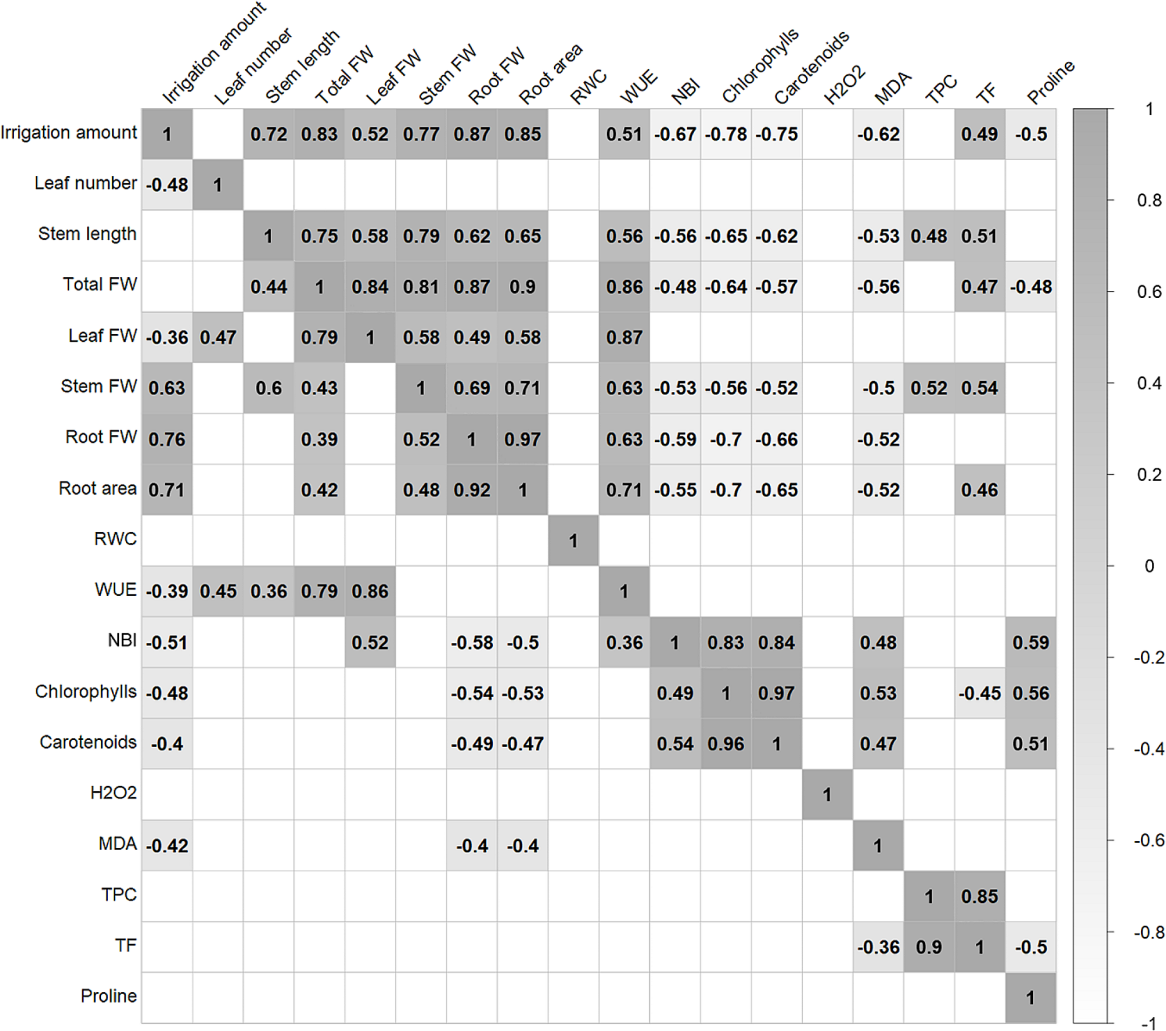
Correlation matrix coefficients for control (upper right diagonal) and water stress (lower left diagonal) in eighteen genotypes evaluated. Only statistically significant correlations (*p*-value < 0.001) are shown

Correlation analysis performed separately for control and water stress conditions showed more significant correlations in the control treatment than the water stress treatment (Fig. 5). In control conditions, all FW traits were positively correlated with each other and with irrigation, stem length, root area and WUE. Stem length and stem FW were positively correlated with TPC, and stem length, total FW and stem DW were positively correlated with TF. Total DW was negatively correlated with proline content, whereas fresh weight traits (except leaf FW) were negatively correlated with NBI, chlorophylls, carotenoids and MDA. Photosynthetic pigments, chlorophylls and carotenoids, were also positively correlated with NBI, MDA and proline. The antioxidant compounds TPC and TF showed a highly positive correlation, but no significant relationship was detected with the oxidative stress markers H_2_O_2_ and MDA (Fig. 5).

Under water stress conditions, total FW was positively correlated with leaf, stem and root FW, root area and WUE. Root growth (root FW and root area) was positively correlated with increased water consumption (irrigation amount) and stem length, and negatively correlated with NBI, chlorophylls, flavonoids and MDA. Irrigation was negatively correlated with FW, WUE, NBI, chlorophylls, carotenoids and MDA. On the other hand, the antioxidant compounds, TF and TPC were strongly positively correlated, and TF was negatively correlated with the oxidative stress marker MDA (Fig. 5).

**Fig. 6:**
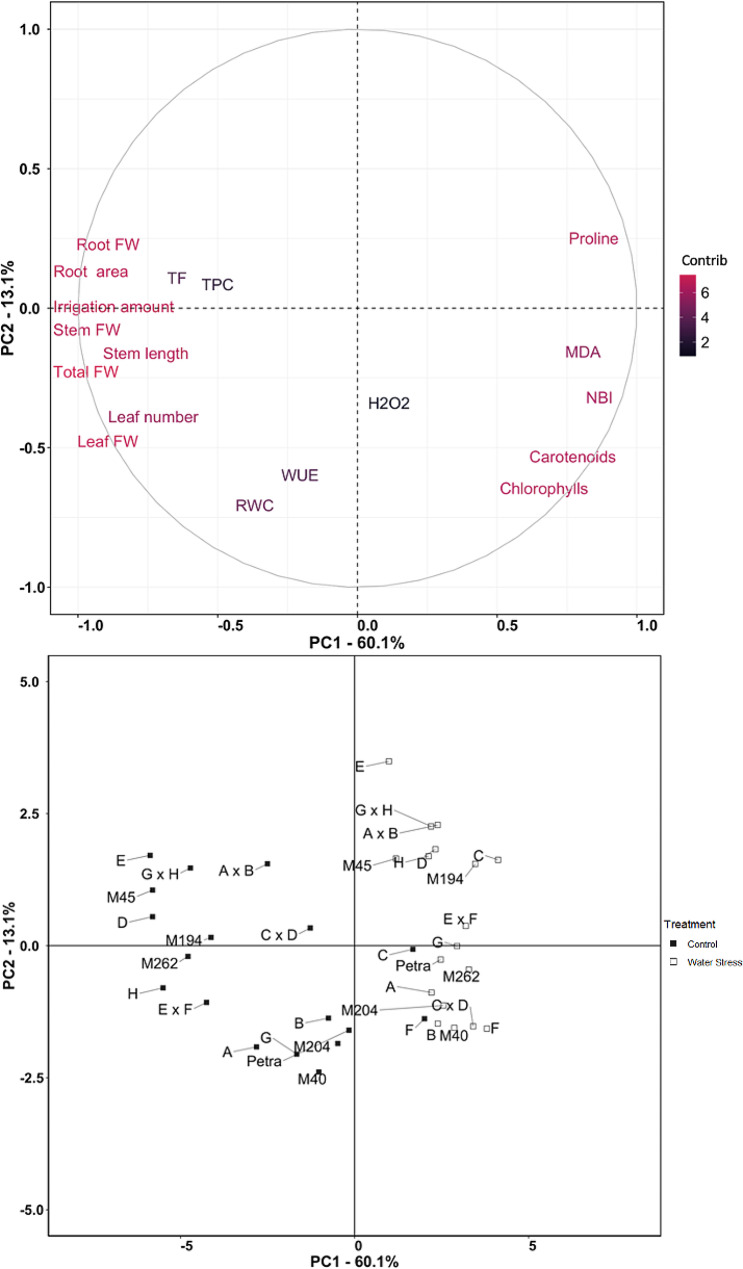
Loading plot (above) and score plot (below) of the principal component analysis (PCA) for the eighteen eggplant genotypes evaluated under control (100% FC) and water stress (30% FC) conditions, based on the first two principal components. The first and second components (PC1 and PC2) represent 60.1% and 13.1% of the variation, respectively

The first two principal components of the principal component analysis (PCA) accounted for 73.2% of the observed variation, with the first principal component (PC1) and the second principal component (PC2) accounting for 60.1% and 13.1% of the variation, respectively (Fig. 6). In the loading plot, PC1 showed high positive correlations (> 0.5) with chlorophylls, carotenoids, NBI, MDA and proline, and high negative correlations (<-0.5) with irrigation, number of leaves, stem length, FW, root area, TF and TPC. On the other hand, PC2 showed high negative correlations (<-0.5) with RWC, WUE, chlorophylls and carotenoids (Fig. 6). In the score plot, the genotypes with negative values for the traits correlated with PC1 under control conditions were located on the first and fourth quadrants of the PCA (negative values for PC1). In contrast, the genotypes with positive values for the traits correlated with PC1 under water stress conditions were located on the second and third quadrants of the PCA, although genotypes C and F grown under control conditions display positive values of PC1 next to the genotypes grown under water stress (Fig. 6).

## Discussion

Breeding for water stress tolerance is challenging due to the complex mechanisms of tolerance [40]. To identify genotypes of interest and use them for breeding, the evaluation of diverse panels of plant genetic resources is essential [41]. In this regard, the eight parental genotypes of the first eggplant MAGIC population, along with their four F_1_ hybrids and a subset of MAGIC S5 recombinant inbred lines, exhibited a high variation and different responses to the water stress conditions for the traits evaluated, revealing genotypic effects in the vast majority of traits evaluated. The observed genetic variability, which leads to varying response to water stress, positions these MAGIC materials as invaluable tools for studying and advancing the genetic enhancement of eggplant for water stress tolerance.

Water stress in plants affects their physiological processes leading to a reduction in growth [42], and, as expected, all genotypes evaluated were affected in their growth when cultivated under water stress conditions. Variation in the response to water deficit was observed amongst the eight MAGIC parents evaluated, indicating variability in drought response mechanisms according to their genetic diversity [43]. Among the traits that allowed survival under water stress conditions, a greater root development, enabling increased water absorption, and a reduced leaf area, limiting transpiration surface, were prominent. These traits were found in parental genotypes D, E and H, which differed significantly from the commercial hybrid Petra; therefore, they could be considered genotypes of interest for breeding eggplant for tolerance to drought. Similarly, Delfin et al. [13] postulate that the best strategy in eggplant against moderate stress is rapid growth with small leaves and a higher allocation to root biomass. However, these traits may result in low productivity in crop plants [44], and further studies are needed to determine the effects on yield. The tolerance response to water stress can be defined as the ability to maintain growth under limited water conditions [45]. In this sense, genotype B did not significantly reduce their growth under water stress and was characterized by their high total FW and leaf FW, which indicates tolerance to water stress. Future experiments on the recovery of stressed plants after irrigating them to 100% FC may provide further relevant information on the capacity of eggplant plants to recover after being subjected to drought stress.

We did not observe a clear effect of water stress on photosynthetic pigments such as chlorophyll and carotenoids, although increases, decreases or stability of their contents have been reported in eggplant when comparing water-stressed plants to well-watered controls [46]. Although most genotypes maintained their values, a lower chlorophyll or carotenoid content was sometimes observed under control conditions. This phenomenon could be attributed to the fact that higher irrigation led to fertiliser dilution, explaining a possible negative correlation between irrigation and chlorophyll, carotenoid and NBI concentrations [10].

At the molecular level, one of the negative effects of water stress on plants is oxidative stress due to increased production of reactive oxygen species, where antioxidant enzymes and compounds are essential to reduce the damage [47]. Two oxidative stress markers were used in this assay: H_2_O_2_, which is not an inducer of oxidative damage but plays an essential role in oxidative signalling [48], and MDA, which increases during lipid oxidation [49]. Thus, eggplant has been shown to increase both peroxidase and MDA levels when exposed to water stress conditions [50]. Amongst the genotypes analysed, G showed an increase in both H_2_O_2_ and MDA, while B, C and D showed an increase in MDA only, indicating a lower tolerance to stress in these genotypes. As a defence mechanism against oxidative stress, plants activate enzymatic and non-enzymatic antioxidant systems that reduce reactive oxygen species (ROS) levels [51]. In this study, the TPC content was evaluated, which did not seem to play an important role as an antioxidant; TF, on the other hand, had an interesting negative correlation with MDA, which could indicate a role as an antioxidant compound in eggplant, in agreement with what has been reported previously [12], where it is observed that tolerant genotypes maintain their MDA levels and increase flavonoids.

Under stressful conditions, a drastic increase in proline content is common in plants, as it functions as an osmoregulatory, signalling and oxidative stress reducer [52]. As observed in this case, proline showed a significant increase when plants were grown under low irrigation conditions, indicating a clear response of the genotypes to the stress treatment. Other studies in eggplant have reported that genotypes with greater tolerance to drought show a greater increase in concentration [12, 53]. Although proline levels were high under water stress conditions, there were no significant differences between genotypes; on the contrary, the basal level under control conditions was much higher in the C parent, representing the wild eggplant (*S. incanum*), than in the other genotypes. However, high proline contents do not seem to be important for non-stressed plants [54]; in our case, even a negative correlation between proline and total FW was observed under control conditions.

Hybrids can result in new genotypes with a superior response, but it is also easy for biological processes to be disrupted, resulting in individuals that perform worse than their parents [55]. In the case of the four hybrids evaluated, the responses were varied and unpredictable, with two hybrids having a high number of traits with a significant increase or decrease in hybrid value. Prediction of polygenic traits, such as those tested in this trial, is usually a major challenge in hybrid generation [56]. These results are similar to those found in other studies that have evaluated F_1_ eggplant hybrids under water stress, where the responses were diverse and complex due to the genetic distance of the parents [16, 57], the generation of hybrids with parents presenting moderate genetic distances could be a better strategy, as it has been seen that they achieve better results in eggplant [58]. At the growth level, the hybrids did not stand out in any trait under water stress, but it was observed that two hybrids had lower H_2_O_2_ content, which could indicate a lower level of oxidative stress [59], and one hybrid had lower proline content, which could indicate a lower level of stress [60]. These are traits that could be of interest for increased drought tolerance. However, the evaluation of a much larger number of genotypes is needed to determine the efficiency of eggplant breeding through hybridization for drought tolerance, as the response of hybrids is difficult to predict [61].

The generation of MAGIC lines has been shown to be a valuable tool for enhancing genetic diversity and developing genotypes with greater adaptation to different environments [62, 63]. The five MAGIC S5 lines evaluated in this study were selected because they were genetically different [21], and this genetic divergence was matched by a different response to water stress. Amongst the lines evaluated, line M45 stood out as tolerant due to its high root growth and low oxidative stress, whereas line M262 showed high susceptibility to stress with low root growth and high oxidative stress. However, no lines were found to be statistically superior to the best parent or to have a large combination of traits favourable for increased tolerance to water stress. Thus, it would be of great interest to evaluate a larger number of lines to search for genotypes with an appropriate combination of traits to maximize tolerance to water stress. With the results found in the tomato MAGIC lines by Diouf et al. [64] on tolerance to abiotic stress, it is to be expected that the evaluation of the complete set of eggplant MAGIC lines will result in the identification of high water-stress tolerant recombinant lines of interest for selection and breeding for resilience.

## Conclusions

Irrigation of eggplant at 30% of field capacity induced water stress in the genotypes evaluated, allowing the analysis of growth and biochemical responses associated with tolerance to water stress. The eight hybrid parents and the S5 MAGIC lines showed a remarkable variability in their responses, making them initial materials of great interest for breeding. Compared to the commercial hybrid Petra, some genotypes showed increased root growth, suggesting potential avenues for improving the eggplant root system. Analysis of photosynthetic pigments showed that increased growth could lead to a reduction in chlorophyll and carotenoid content. With regard to oxidative stress, it was observed that flavonoid levels might be involved in mitigating the adverse effects of water stress, suggesting that selection for this trait may be of interest for drought tolerance in eggplant. Concerning the F_1_ hybrids, diverse and unpredictable responses were observed, with heterosis manifesting both positively and negatively in the traits evaluated. The genetic diversity in the MAGIC lines resulted in a wide range of responses to tolerance, highlighting the diversity of traits present. Our results indicated a considerable variation in the MAGIC materials evaluated for tolerance to water stress, which is of interest for breeding new resilient eggplant cultivars. Screening larger sets of MAGIC lines may result in the identification of recombinant lines with increased tolerance to water stress.

## Data Availability

The datasets used and/or analysed during the current study are available from the corresponding author upon reasonable request.

## References

[1] Calanca PP, Ahmed M, Stockle CO (2017). Effects of Abiotic stress in Crop Production. Quantification of Climate Variability, Adaptation and Mitigation for Agricultural sustainability.

[2] Hanks RJ, Rasmussen VP. Predicting Crop Production as Related to Plant Water Stress. In: Brady NC, editor. Advances in Agronomy. Academic Press. 1982;35:193–215.

[3] Yadav S, Modi P, Dave A, Vijapura A, Patel D, Patel M et al. Effect of Abiotic Stress on Crops. In: Sustainable Crop Production. IntechOpen. 2020.

[4] Gany AHA, Sharma P, Singh S (2019). Global review of Institutional reforms in the Irrigation Sector for Sustainable Agricultural Water Management, including water users’ associations. Irrig Drain.

[5] Jacobsen SE, Jensen CR, Liu F (2012). Improving crop production in the arid Mediterranean climate. Field Crops Res.

[6] Mancosu N, Snyder RL, Kyriakakis G, Spano D (2015). Water Scarcity and Future challenges for Food Production. Water.

[7] Shahzad A, Ullah S, Dar AA, Sardar MF, Mehmood T, Tufail MA (2021). Nexus on climate change: agriculture and possible solution to cope future climate change stresses. Environ Sci Pollut Res.

[8] Garcia-Caparros P, Contreras JI, Baeza R, Segura ML, Lao MT (2017). Integral Management of Irrigation Water in Intensive Horticultural systems of Almería. Sustainability.

[9] Dumitru EA, Berevoianu RL, Tudor VC, Teodorescu FR, Stoica D, Giucă A (2023). Climate Change impacts on Vegetable crops: a systematic review. Agriculture.

[10] Díaz-Pérez JC, Eaton TE (2015). Eggplant (*Solanum melongena* L.) Plant Growth and Fruit Yield as affected by drip irrigation rate. HortScience.

[11] Amiri Rodan M, Hassandokht MR, Sadeghzadeh-Ahari D, Mousavi A (2020). Mitigation of drought stress in eggplant by date straw and plastic mulches. J Saudi Soc Agricultural Sci.

[12] Plazas M, González-Orenga S, Nguyen HT, Morar IM, Fita A, Boscaiu M (2022). Growth and antioxidant responses triggered by water stress in wild relatives of eggplant. Sci Hortic.

[13] Delfin EF, Drobnitch ST, Comas LH (2021). Plant strategies for maximizing growth during water stress and subsequent recovery in *Solanum melongena* L. (eggplant). PLoS ONE.

[14] Flores-Saavedra M, Gramazio P, Vilanova S, Mircea DM, Ruiz-González MX, Vicente O et al. Introgressed eggplant lines with the wild *Solanum incanum* evaluated under drought stress conditions. J Integr Agric. 2024.

[15] Yu D, Gu X, Zhang S, Dong S, Miao H, Gebretsadik K (2021). Molecular basis of heterosis and related breeding strategies reveal its importance in vegetable breeding. Hortic Res.

[16] González-Orenga S, Plazas M, Ribera E, Pallotti C, Boscaiu M, Prohens J (2023). Transgressive Biochemical Response to water stress in Interspecific Eggplant hybrids. Plants.

[17] Arrones A, Vilanova S, Plazas M, Mangino G, Pascual L, Díez MJ (2020). The Dawn of the age of multi-parent MAGIC populations in plant breeding: novel powerful Next-Generation resources for genetic analysis and selection of recombinant Elite Material. Biology.

[18] Rida S, Maafi O, López-Malvar A, Revilla P, Riache M, Djemel A (2021). Genetics of Germination and Seedling traits under Drought stress in a MAGIC Population of Maize. Plants.

[19] Diaz S, Ariza-Suarez D, Izquierdo P, Lobaton JD, de la Hoz JF, Acevedo F (2020). Genetic mapping for agronomic traits in a MAGIC population of common bean (*Phaseolus vulgaris* L.) under drought conditions. BMC Genomics.

[20] Thudi M, Samineni S, Li W, Boer MP, Roorkiwal M, Yang Z et al. Whole genome resequencing and phenotyping of MAGIC population for high resolution mapping of drought tolerance in chickpea. Plant Genome. 2023;e20333. 10.1002/tpg2.20333 PMC12806880 37122200

[21] Mangino G, Arrones A, Plazas M, Pook T, Prohens J, Gramazio P (2022). Newly developed MAGIC Population allows identification of strong associations and candidate genes for anthocyanin pigmentation in Eggplant. Front Plant Sci.

[22] Ranil RHG, Niran HML, Plazas M, Fonseka RM, Fonseka HH, Vilanova S (2015). Improving seed germination of the eggplant rootstock *Solanum torvum* by testing multiple factors using an orthogonal array design. Sci Hortic.

[23] Hoagland DR, Arnon DI (1950). The water-culture method for growing plants without soil. Calif Agricultural Exp Stn Circular.

[24] Rolando JL, Ramírez DA, Yactayo W, Monneveux P, Quiroz R (2015). Leaf greenness as a drought tolerance related trait in potato (*Solanum tuberosum* L). Environ Exp Bot.

[25] Seethepalli A, Dhakal K, Griffiths M, Guo H, Freschet GT, York LM (2021). RhizoVision Explorer: open-source software for root image analysis and measurement standardization. AoB Plants.

[26] Cerovic ZG, Cartelat A, Goulas Y, Meyer S. In-field assessment of wheat-leaf polyphenolics using the new optical leaf-clip Dualex. In: Precision Agriculture ‘05. Wageningen Academic. 2005;243–250.

[27] Lichtenthaler HK, Wellburn AR (1983). Determinations of total carotenoids and chlorophylls a and b of leaf extracts in different solvents. Biochem Soc Trans.

[28] Loreto F, Velikova V (2001). Isoprene produced by leaves protects the photosynthetic apparatus against ozone damage, quenches ozone products, and reduces lipid peroxidation of Cellular membranes. Plant Physiol.

[29] Hodges DM, DeLong JM, Forney CF, Prange RK (1999). Improving the thiobarbituric acid-reactive-substances assay for estimating lipid peroxidation in plant tissues containing anthocyanin and other interfering compounds. Planta.

[30] Blainski A, Lopes GC, De Mello JCP (2013). Application and analysis of the Folin Ciocalteu Method for the determination of the total phenolic content from *Limonium brasiliense* L. Molecules.

[31] Zhishen J, Mengcheng T, Jianming W (1999). The determination of flavonoid contents in mulberry and their scavenging effects on superoxide radicals. Food Chem.

[32] Bates LS, Waldren RP, Teare ID (1973). Rapid determination of free proline for water-stress studies. Plant Soil.

[33] Di Renzo JA, Casanoves F, Balzarini MG, Gonzalez L, Tablada M, Robledo C. Infostat - Software estadístico. 2020.

[34] Geng X, Qu Y, Jia Y, He S, Pan Z, Wang L (2021). Assessment of heterosis based on parental genetic distance estimated with SSR and SNP markers in upland cotton (*Gossypium hirsutum* L). BMC Genomics.

[35] Mangino G, Plazas M, Vilanova S, Prohens J, Gramazio P (2020). Performance of a set of eggplant (*Solanum melongena*) Lines with introgressions from its wild relative *S. incanum* under Open Field and Screenhouse conditions and detection of QTLs. Agronomy.

[36] R. A language and environment for statistical computing (Versión 4.2.1) [Software]. R Foundation for Statistical Computing. R Core Team; 2022.

[37] Revelle W. psych: Procedures for Psychological, Psychometric, and Personality Research. 2023.

[38] Wei T, Simko V. R package ‘corrplot’: Visualization of a Correlation Matrix (Version 0.92). 2021.

[39] Wickham H. Elegant Graphics for Data Analysis. 2016.

[40] Witcombe JR, Hollington PA, Howarth CJ, Reader S, Steele KA (2007). Breeding for abiotic stresses for sustainable agriculture. Philos Trans R Soc B Biol Sci.

[41] Fu YB (2015). Understanding crop genetic diversity under modern plant breeding. Theor Appl Genet.

[42] Blum A, Blum A (2011). Plant Water relations, Plant Stress and Plant Production. Plant breeding for Water-Limited environments.

[43] Gramazio P, Yan H, Hasing T, Vilanova S, Prohens J, Bombarely A (2019). Whole-genome resequencing of seven eggplant (*Solanum melongena*) and one wild relative (*S. Incanum*) Accessions provides new insights and breeding tools for Eggplant Enhancement. Front Plant Sci.

[44] Farooq M, Hussain M, Wahid A, Siddique KHM, Aroca R (2012). Drought stress in plants: an overview. Plant responses to Drought stress: from morphological to molecular features.

[45] Salehi-Lisar SY, Bakhshayeshan-Agdam H, Hossain MA, Wani SH, Bhattacharjee S, Burritt DJ, Tran LSP (2016). Drought stress in plants: causes, consequences, and Tolerance. Drought stress tolerance in plants, vol 1: physiology and Biochemistry.

[46] Flores-Saavedra M, Plazas M, Vilanova S, Prohens J, Gramazio P (2023). Induction of water stress in major Solanum crops: a review on methodologies and their application for identifying drought tolerant materials. Sci Hortic.

[47] Hasanuzzaman M, Nahar K, Gill SS, Fujita M. Drought stress responses in plants, oxidative stress, and antioxidant defense. Climate change and plant abiotic stress tolerance. John Wiley & Sons, Ltd.; 2013. pp. 209–50.

[48] Jubany-Marí T, Munné-Bosch S, Alegre L (2010). Redox regulation of water stress responses in field-grown plants. Role of hydrogen peroxide and ascorbate. Plant Physiol Biochem.

[49] Anjum NA, Sofo A, Scopa A, Roychoudhury A, Gill SS, Iqbal M (2015). Lipids and proteins—major targets of oxidative modifications in abiotic stressed plants. Environ Sci Pollut Res.

[50] Hannachi S, Signore A, Adnan M, Mechi L (2022). Single and Associated effects of Drought and Heat stresses on physiological, biochemical and antioxidant Machinery of Four Eggplant cultivars. Plants.

[51] Hasanuzzaman M, Hossain MA, da Silva JAT, Fujita M, Venkateswarlu B, Shanker AK, Shanker C, Maheswari M (2012). Plant Response and Tolerance to Abiotic oxidative stress: antioxidant defense is a key factor. Crop stress and its management: perspectives and strategies.

[52] Kaur G, Asthir B (2015). Proline: a key player in plant abiotic stress tolerance. Biol Plant.

[53] Plazas M, Nguyen HT, González-Orenga S, Fita A, Vicente O, Prohens J (2019). Comparative analysis of the responses to water stress in eggplant (*Solanum melongena*) cultivars. Plant Physiol Biochem.

[54] Hayat S, Hayat Q, Alyemeni MN, Wani AS, Pichtel J, Ahmad A (2012). Role of proline under changing environments. Plant Signal Behav.

[55] Bar-Zvi D, Lupo O, Levy AA, Barkai N (2017). Hybrid vigor: the best of both parents, or a genomic clash?. Curr Opin Syst Biol.

[56] Riedelsheimer C, Czedik-Eysenberg A, Grieder C, Lisec J, Technow F, Sulpice R (2012). Genomic and metabolic prediction of complex heterotic traits in hybrid maize. Nat Genet.

[57] Kouassi AB, Kouassi KBA, Sylla Z, Plazas M, Fonseka RM, Kouassi A (2021). Genetic parameters of drought tolerance for agromorphological traits in eggplant, wild relatives, and interspecific hybrids. Crop Sci.

[58] Rajan N, Debnath S, Perveen K, Khan F, Pandey B, Srivastava A (2023). Optimizing hybrid vigor: a comprehensive analysis of genetic distance and heterosis in eggplant landraces. Front Plant Sci.

[59] Černý M, Habánová H, Berka M, Luklová M, Brzobohatý B (2018). Hydrogen peroxide: its role in Plant Biology and Crosstalk with Signalling Networks. Int J Mol Sci.

[60] Parkash V, Singh S (2020). A review on potential plant-based water stress indicators for Vegetable crops. Sustainability.

[61] Fritsche-Neto R, Galli G, Borges KLR, Costa-Neto G, Alves FC, Sabadin F (2021). Optimizing genomic-enabled prediction in Small-Scale Maize Hybrid Breeding Programs: a Roadmap Review. Front Plant Sci.

[62] Li XF, Liu ZX, Lu DB, Liu YZ, Mao XX, Li ZX (2013). Development and evaluation of multi-genotype varieties of rice derived from MAGIC lines. Euphytica.

[63] Samineni S, Sajja SB, Mondal B, Chand U, Thudi M, Varshney RK (2021). MAGIC lines in chickpea: development and exploitation of genetic diversity. Euphytica.

[64] Diouf IA, Derivot L, Bitton F, Pascual L, Causse M (2018). Water deficit and salinity stress reveal many specific QTL for Plant Growth and Fruit Quality traits in Tomato. Front Plant Sci.

